# A Balanced IL-1β Activity Is Required for Host Response to *Citrobacter rodentium* Infection

**DOI:** 10.1371/journal.pone.0080656

**Published:** 2013-12-02

**Authors:** Misagh Alipour, Yuefei Lou, Daniel Zimmerman, Michael W. Bording-Jorgensen, Consolato Sergi, Julia J. Liu, Eytan Wine

**Affiliations:** 1 Department of Pediatrics, University of Alberta, Edmonton, Alberta, Canada; 2 Centre of Excellence for Gastrointestinal Inflammation and Immunity Research (CEGIIR), University of Alberta, Edmonton, Alberta, Canada; 3 Department of Pathology, University of Alberta, Edmonton, Alberta, Canada; 4 Department of Medicine, University of Alberta, Edmonton, Alberta, Canada; Duke University Medical Center, United States of America

## Abstract

Microbial sensing plays essential roles in the innate immune response to pathogens. In particular, NLRP3 forms a multiprotein inflammasome complex responsible for the maturation of interleukin (IL)-1β. Our aim was to delineate the role of the NLRP3 inflammasome in macrophages, and the contribution of IL-1β to the host defense against *Citrobacter rodentium* acute infection in mice. *Nlrp3^−/−^* and background C57BL/6 (WT) mice were infected by orogastric gavage, received IL-1β (0.5 µg/mouse; ip) on 0, 2, and 4 days post-infection (DPI), and assessed on 6 and 10 DPI. Infected *Nlrp3^−/−^* mice developed severe colitis; IL-1β treatments reduced colonization, abrogated dissemination of bacteria to mesenteric lymph nodes, and protected epithelial integrity of infected *Nlrp3^−/−^* mice. In contrast, IL-1β treatments of WT mice had an opposite effect with increased penetration of bacteria and barrier disruption. Microscopy showed reduced damage in *Nlrp3^−/−^* mice, and increased severity of disease in WT mice with IL-1β treatments, in particular on 10 DPI. Secretion of some pro-inflammatory plasma cytokines was dissipated in *Nlrp3^−/−^* compared to WT mice. IL-1β treatments elevated macrophage infiltration into infected crypts in *Nlrp3^−/−^* mice, suggesting that IL-1β may improve macrophage function, as exogenous administration of IL-1β increased phagocytosis of *C. rodentium* by peritoneal *Nlrp3^−/−^* macrophages *in vitro*. As well, the exogenous administration of IL-1β to WT peritoneal macrophages damaged the epithelial barrier of *C. rodentium*-infected polarized CMT-93 cells. Treatment of *Nlrp3^−/−^* mice with IL-1β seems to confer protection against *C. rodentium* infection by reducing colonization, protecting epithelial integrity, and improving macrophage activity, while extraneous IL-1β appeared to be detrimental to WT mice. Together, these findings highlight the importance of balanced cytokine responses as IL-1β improved bacterial clearance in *Nlrp3^−/−^* mice but increased tissue damage when given to WT mice.

## Introduction

The non-invasive Gram-negative bacterium *Citrobacter rodentium* is a natural mouse pathogen, commonly used for the study of enteric infections and bacteria-induced inflammation as it resembles enteropathogenic and enterohaemorrhagic *Escherichia coli* infections in humans [1]. Oral transmission of *C. rodentium* in mice initiates with the passage through the cecum, followed by intimate colonization to the colonic epithelium lining, through formation of attaching and effacing (A/E) lesions [2], [3]. This adhesion leads to the destruction of brush-border microvilli, epithelial cell hyperplasia, and goblet cell depletion [4]. Multiple factors, including the genetic background and age of the mouse will determine its susceptibility, ranging from self-limited colitis to fatality [5], [6]. Ultimately, an aggressive adaptive immune response over the course of 2 to 4 weeks can clear the infection and provide immunity to future challenge [7], [8].

Innate immune responses, and in particular gut resident macrophages, play essential roles in the early stages of response to *C. rodentium* infection [9]–[11]. Transmembrane Toll-like receptors-2 (TLR-2) and TLR-4, the signaling adaptor protein myeloid differentiation factor (MyD)-88, and nuclear factor-kappa B (NF-κB) mediate the inflammatory response to *C. rodentium* by recruiting macrophages and neutrophils through the induction of chemokines [12]–[16]. Other regulators of intestinal homeostasis and epithelial integrity include the cytosolic nucleotide-binding oligomerization domain (NOD) and the NOD-like receptor (NLR) family expressed in epithelial cells and macrophages [11], [17]. Mice lacking NOD1 or NOD2 are impaired in *C. rodentium* clearance with classical signs of inflammation and dissemination [18].

In particular, the macrophage NLRP3 protein, which is activated by a plethora of stimuli, was recently shown to be a key component in the immune response to *C. rodentium* [19]. In presence of various microbial stimuli, including *C. rodentium*, the NLRP3 protein oligomerizes and recruits multiple protein domains to form the NLRP3 inflammasome [20], [21]. Although it remains unclear how *C. rodentium* activates NLRP3 inflammasome, this triggers procaspase-1 dimerization and self-activation, which then processes the maturation of cellular interleukins (IL) pro-IL-1β and pro-IL-18 to the active cytokines, leading to their secretion by an unknown pathway [22], [23]. As well, negative regulation of caspase-1 through caspase-12 leads to hyperproduction of IL-1β and IL-18 in macrophages [24]. Interestingly, *C. rodentium* can also induce caspase-1-dependent IL-1β maturation and secretion through a synergistic TLR-4 and NLRP3 pathway *in vivo* [25], [26]. The secretion of IL-1β leads to further production of pro-IL-1β through the IL-1 receptor as well as cascading to a pro-inflammatory immune response with an influx of inflammatory cells to the site of infection [27], [28].

Previously, it was established that mice lacking the *Nlrp3* gene were more susceptible to induced experimental colitis [29], and *Nlrp3^−/−^* macrophages did not respond to pathogen-associated microbial patterns [5]. Moreover, mice lacking the NLRP3 inflammasome, caspase-1, or its cytokines (IL-1β and IL-18), had delayed *C. rodentium* clearance; however the NLRP3 inflammasome was specifically required to induce IL-1β production in macrophages [19], [30]. As well, the IL-1 receptor signaling through the macrophage NLRP3 inflammasome appeared to be required for *C. rodentium* clearance [31].

This study was conducted to delineate the intriguing role of the IL-1β cytokine and the macrophage NLRP3 inflammasome in an acute *C. rodentium* infection. Our findings indicate that compensation of IL-1β in mice lacking the NLRP3 inflammasome may promote the clearance of *C. rodentium* by stimulating inflammatory macrophages in the early stages of infection. Conversely, IL-1β overcompensation may be detrimental in WT mice.

## Materials and Methods

### Animal care and ethics statement

Groups consisting of seven to eight week-old male and female mice (n = 6 to 8 per group) were used. C57BL/6 (WT) mice were purchased from Charles River Canada (Montreal, QC, Canada), and *Nlrp3^−/−^* mice on the C57BL/6 background (generously provided by Dr. Daniel Muruve, University of Calgary, AB, Canada) were bred and housed in a Canadian Council on Animal Care (CCAC) accredited research animal facility at the University of Alberta. All animals used in the study were treated and cared for in accordance with the guidelines recommended by the CCAC. The experimental protocol was reviewed and approved by the Animal Care and Use Committee for Health Sciences at the University of Alberta (#661). The animals were kept at room temperature (RT) 20–22°C, relative humidity 30–70%, exposed to alternate cycles of 12 h light and darkness cages, and with free access to irradiated pelleted laboratory chow and sterilized tap water *ad libitum*. Access to food or water was not disrupted prior to infection.

### *C. rodentium* infection and study design

Fresh bacteria were prepared by inoculating colonies of *C. rodentium* DBS100 (ATCC 51459) from Lysogeny agar plates into 10 ml of Lysogeny broth (LB) overnight at 37°C under static conditions. Cultures were centrifuged and resuspended to approximately 1×10^9^ colony forming unit (CFU) per ml in sterile 0.9% saline using a spectrophotometer (optical density at 600 nm) and by plating. Mice were oro-gavaged with 100 µl of the *C. rodentium* suspension (approximately 1×10^8^ CFU/mouse) or sterile 0.9% saline with a non-flexible blunt-ended stainless steel animal feeding needle. The mice were returned to their cages and weights were recorded every two days; fecal colonization was documented by rectal swabbing and typical growth of pink colonies on MacConkey agar plates (37°C for 24 h), indicative of *C. rodentium*. Colonies were confirmed to be *C. rodentium* by real-time polymerase chain reaction (RT-PCR) [8] (Appendix S1).

On 0, 2, and 4 days post-infection (DPI), assigned groups received lyophilized recombinant mouse IL-1β (0.5 µg/mouse ip; BioLegend, San Diego, USA) or equal volumes of sterile injectable 0.9% saline. IL-1β dosage was based on a previous *in vivo* infection model [32]. On 6 and 10 DPI, mice were anesthetized by isoflurane (Halocarbon Products; River Edge, NJ, USA) and euthanized by cervical dislocation.

### Bacterial colonization and translocation

Fecal content of the cecum was weighed and suspended in 1 ml ice-cold sterile phosphate buffered saline (PBS) buffer. Colons were flushed with PBS to remove stool, trimmed free of mesenteric fat and cut longitudinally. A portion from proximal to distal colon was weighed and homogenized in 1 ml PBS. The homogenizer was rinsed with double distilled water, then ethanol, and rinsed with PBS, a step performed twice between samples to avoid bacterial contamination of samples. Mesenteric lymph nodes (MLN) were removed, weighed, and homogenized (PowerGen Model 125, Fisher Scientific) in 1 ml PBS. The cecum fecal content, and colon and MLN homogenates, were serially diluted and plated on MacConkey agar, and incubated aerobically (37°C for 24 h). Colonies were counted on the highest diluted plate with a maximum of 300 CFU/plate, with the lower limit of detection being 1 bacterium due to a 10-fold dilution factor, corresponding to 10 bacteria. Colony counts per mg tissue were calculated by multiplying CFU/plate by the dilution and then dividing by tissue weight.

### Intestinal integrity

Colonic epithelial-layer integrity was assessed as described previously [33] using EZ-link Sulfo-NHS-Biotin (Pierce Chemical; Rockford, IL, USA) as a molecular tracer. In brief, immediately after sacrifice, the abdomen was opened and the distal colon cut. Biotin (2 mg/ml in PBS) was slowly administered into the colon *via* syringe attached to a 22G blunt-end needle for 3 min. A 1-cm region above the injection site (distal colon) was removed and placed into 4% paraformaldehyde (PFA) in PBS (pH 7.3; 3 h) and rocked gently. The tissue was washed thrice in PBS and immersed in optimal cutting temperature media (Tissue-Tek) and frozen (−80°C). Cryosections (5 µm) were fixed in ice-cold acetone for 5 min, then blocked with blocking solution [2% goat serum and 1% fetal bovine serum (FBS)] and stained with rabbit anti-*Citrobacter* polyclonal antibody (a kind gift from Dr. Philip M. Sherman, Hospital for Sick Children, Toronto, ON, Canada) (1∶300 dilution, overnight, 4°C), followed by a secondary anti-rabbit Alexa-546 (1∶500 dilution, 1 h, RT; Life Technologies, Burlington, ON, Canada). Then, for biotin secondary staining, the sections were incubated with streptavidin conjugated to Alexa-488 (1∶500 dilution, 30 min, RT; Life Technologies), followed by cellular DNA 4′,6-diamidino-2-phenylindole (DAPI) counterstaining (5 µg/ml, 1 min, RT). The slides were imaged with an AxioCam ERc 5s camera at X400 magnification (Zeiss AX10 Observer.Z1 inverted microscope; Carl Zeiss Canada Ltd., Toronto, ON, Canada) and analyzed using the ZEN 2012 software.

### Histopathological scoring and crypt cell hyperplasia

Colon tissues were “swiss-rolled”, fixed in 10% phosphate-buffered formalin, dehydrated by a tissue processor and embedded in paraffin. Tissues were sectioned using a microtome (5 µm) and H&E stained with an autostainer (Autostainer XL, Leica Microsystems; Concord, ON, Canada). Histological scoring of coded slides was evaluated by a single pathologist (CS), blinded to the source of the slides, based on a modified system as previously described [34]. The parameters included: goblet cells, mucosal thickening, inflammatory cells, submucosa cell infiltration, destruction of architecture, epithelial surface ulcers, crypt abscesses, and eosinophil infiltration, each receiving a score of 0–4. To quantify epithelial cell hyperplasia, 5 sections of each mouse colon containing a minimum of 5 well-oriented crypts (>25 crypts per mouse) were imaged at X200 magnification (Zeiss microscope). The length was measured from the base of the crypt to the mucosal surface using the ZEN 2012 software by an individual blinded to the origin of each slide. Cellular proliferation was also determined by proliferating cell nuclear antigen (PCNA) staining (Appendix S1).

### Cytokine analysis

Plasma samples (0.6 mL) were collected immediately after sacrifice by cardiac puncture in Vacutainer plasma tubes, centrifuged and plasma stored at −80°C. Colon sections were stored in M-PER reagent (Thermo Scientific; Rockford, IL, USA) and frozen at −80°C. At the time of analysis, tissues were homogenized on ice by sonication, centrifuged and the supernatant used immediately. The concentrations of plasma and colon tissue cytokines and chemokines were determined using a mouse pro-inflammatory multiplex kit according to the manufacturer's protocol, measuring IL-6, IL-12p70, tumor necrosis factor (TNF)-α, IL-1β, KC/GRO, and interferon (IFN)-γ (Meso Scale Discovery; Gaithersburg, MD, USA). The colon tissue cytokine and chemokine concentrations of IL-17, IL-22, and monocyte chemoattractant protein-1 (MCP-1) were determined using singleplex kits according to the manufacturer's protocol (Meso Scale Discovery). Colon tissue concentrations were normalized to the protein concentration (determined using a Bradford protein assay).

### Colon tissue macrophage staining

Cryo-fixed sections from the distal colon were used for detection of macrophages and their proximity to the *C. rodentium* colonization in the crypts. The sections were stained for *C. rodentium* and then secondary anti-rabbit Alexa-488 (1∶500 dilution, 30 min, RT) as described above. For macrophage staining, sections were incubated with rat anti-macrophage F4/80 (1∶200 dilution, overnight, 4°C) (AbD Serotec; Raleigh, NC, USA), followed by secondary anti-rat Alexa-594 (1∶400 dilution, 1 h, RT; Life Technologies). Slides were counterstained with DAPI (5 µg/ml, 1 min, RT) and images were taken at X200 and X630 magnification (Zeiss microscope).

### *In vitro* infection, immunofluorescence, and western blotting

Peritoneal WT and *Nlrp3^−/−^* macrophages were isolated and seeded in 24-well plates (Appendix S1) [35]. Macrophages were then induced by IL-1β (10 ng/ml final concentration) in the medium 1 h prior to *C. rodentium* infection (100∶1 multiplicity of infection). After 1 h of infection, cells were washed thrice with PBS. To quantify intracellular (phagocytosed) bacteria, gentamicin (100 µg/ml) was added for 1 h to kill extracellular bacteria; macrophages were then lysed in 1% Triton X-100 for 15 min, and serially plated for enumeration. To complement this approach, macrophages and phagocytosed *C. rodentium* were stained and imaged. In brief, infected macrophages (grown on 13 mm glass slides and following the procedure detailed above) were fixed in 4% PFA for 15 min at RT and blocked for 1 h, then permeabilized with 0.2% Triton X-100 (10 min). Slides were stained for macrophages, *C. rodentium*, and DAPI as described above, and imaged at X630 magnification (Zeiss microscope). Western blotting of the macrophages and supernatant were performed to measure the secretion of mature IL-1β and the absence of mature IL-1β in *Nlrp3^−/−^* macrophages (Appendix S1).

### Epithelial barrier assessment

The role of macrophages and IL-1β in the maintenance of the intestinal barrier in presence of *C. rodentium* was monitored *in vitro* in real-time using the Electric Cell–substrate Impedance Sensing 1600R device (ECIS; Applied BioPhysics; Troy, NY, USA) by measuring resistance at 4 kHz. Mouse colorectal epithelial CMT-93 cells (grown in DMEM-F12, supplemented with 5% FBS) were seeded on sterile 8-well gold-plated electrode arrays (8W10E PET) at 5×10^5^ cells/ml. The arrays were incubated at 37°C, 5% CO_2_, and resistance continuously recorded. At 24 h, when the polarized cells formed a barrier, indicated by plateauing of the transepithelial electrical resistance (TER), CMT-93 cells were washed and medium without antibiotics was added. Cells were inoculated with *C. rodentium* (20∶1 multiplicity of infection) or left uninfected. After 1 h, the unadhered bacteria were aspirated, and replaced with medium containing 5 µg/ml gentamicin to inhibit *C. rodentium* overgrowth. Isolated WT and *Nlrp3^−/−^* peritoneal macrophages (Appendix S1) were inoculated on selected wells (10∶1 ratio to initial epithelial cells seeded). Exogenous IL-1β (50 ng/ml final concentration) was then inoculated on selected wells. Controls, including epithelial cells with or without macrophages or IL-1β, were also included. Resistance (ohms) were continuously collected by the 1600R device software for another 18 h and normalized to the time point 1 h after the macrophage/IL-1β inoculation. Data represent two independent experiments.

### Data analysis

Results were expressed as mean ± standard error (SE). The statistical analyses were performed using InStat 3.0 (GraphPad Software, San Diego, CA, USA). Comparisons between the means of 2 groups were made by an unpaired non-parametric two-tailed Mann-Whitney test. Comparisons between the means of 3 or more groups were made with the non-parametric Kruskal-Wallis analysis of variance (ANOVA) and Dunn's multiple comparison tests. Significance: one asterisk: *P*<0.05, two asterisks: *P*<0.01, and three asterisks: *P*<0.001.

## Results

### IL-1*β* promotes *C. rodentium* clearance and maintains intestinal integrity in *Nlrp3^−/−^* mice

**Figure 1:**
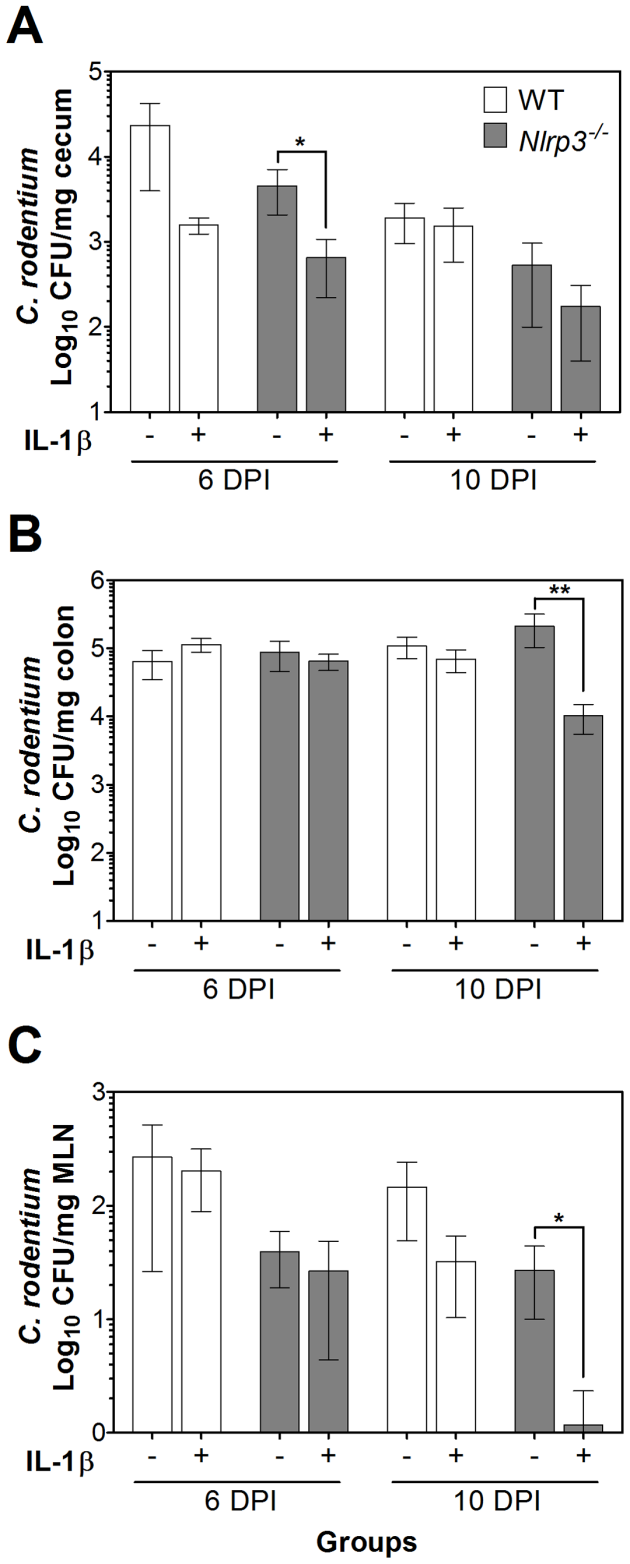
IL-1β treatments reduce intestinal colonization and dissemination of *C. rodentium* in *Nlrp3^−/−^* mice. Infected mice were given repeated IL-1β treatments on 0, 2, and 4 DPI. *C. rodentium* colonization of the (A) cecum, (B) colon, and (C) dissemination to MLN were assessed on 6 and 10 DPI by plating homogenates on MacConkey agar plates in serial dilutions. IL-1β treatments of *Nlrp3^−/−^* mice significantly lowered cecum colonization on 6 DPI, and colon colonization and dissemination to MLN on 10 DPI. Values were Log_10_ transformed and data presented as mean ± SE. One asterisk: *P*<0.05, two asterisks: *P*<0.01.

Mice were infected orally with *C. rodentium*. On 0, 2, and 4 DPI, the mice were given recombinant active IL-1β by intraperitoneal injection to stimulate macrophage response. All infected mice were confirmed to have *C. rodentium* in their stool by 4 DPI. On 6 and 10 DPI, IL-1β treatments of *Nlrp3^−/−^* mice reduced cecum (*P*<0.05) and colon (*P*<0.01) colonization, respectively (**Figure 1A and 1B**). Multiple colonies from positive MacConkey agar plates plated with homogenized MLN were confirmed to be *C. rodentium* by RT-PCR (data not shown), indicating barrier breakdown and bacterial translocation to MLN. The rates of dissemination of *C. rodentium* to MLN were significantly reduced in IL-1β treated *Nlrp3^−/−^* mice (*P*<0.05, **Figure 1C**).

### IL-1*β* treatments improve integrity of the epithelial barrier in infected *Nlrp3^−/−^* mice

**Figure 2:**
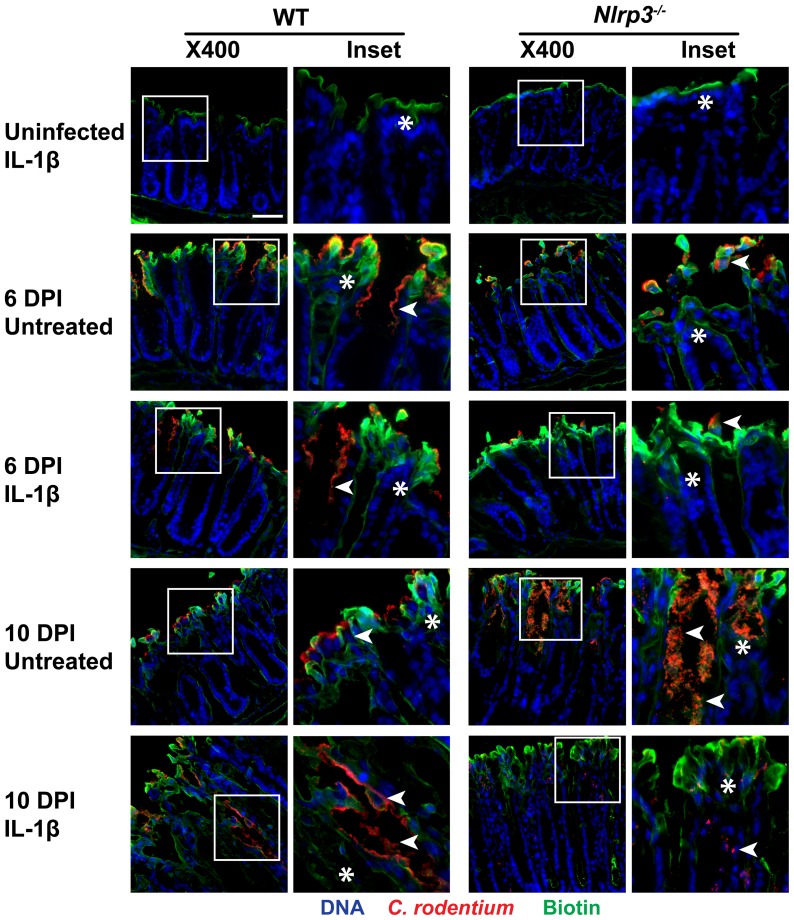
IL-1β treatments modulate *C. rodentium* penetration and epithelial integrity. Biotin was administered into the lumen of the distal colon at 6 or 10 DPI. Cryosections were stained and viewed under fluorescent microscopy for *C. rodentium* infiltration (in red; indicated by arrowheads in the enlarged image of the inset highlighted by boxes in the left panels) and biotin penetration (green; indicated by asterisks). Uninfected IL-1β-treated mice retained biotin to the apical surface. In non-treated mice at 6 DPI, the bacterial infiltration and biotin penetration was increased. IL-1β treatments at 6 DPI did not seem to have a major effect on infiltration or epithelial integrity. In non-treated mice at 10 DPI, the bacterial infiltration and epithelial barrier disruption were lowered in WT and elevated in *Nlrp3^−/−^* mice, relative to 6 DPI. In IL-1β-treated mice at 10 DPI, the bacterial infiltration and barrier disruption were elevated in WT and lowered in *Nlrp3^−/−^* mice. Bacterial colonization followed the same trend as biotin penetration. Magnification X400, bar 50 µm.

To assess the integrity of colonic epithelia and the possible protection conferred by IL-1β, biotin was administered into the colonic lumen of the mice at 6 and 10 DPI, and fluorescently labeled to demonstrate how deep a macromolecule could permeate through the injured mucosa. As well, to visualize *C. rodentium* adhesion to, and infiltration through the epithelial surface, anti-*Citrobacter* antibody was used. This method enables simultaneous visualization of bacteria penetration and barrier disruption to assess possible correlations between infection and permeability. As shown on **Figure 2**, biotin penetration was retained to the apical surface in IL-1β-treated uninfected mice. Biotin penetrated the epithelial surface in infected mice at 6 and 10 DPI, specifically in regions of high *C. rodentium* adhesion and in areas where bacteria seemed to invade into the lamina propria. *C. rodentium* propagation to the lamina propria appeared higher at 10 DPI in *Nlrp3^−/−^* mice than in WT. However, the IL-1β treatments tended to protect epithelial integrity and lowered *C. rodentium* adhesion to crypts by 10 DPI in *Nlrp3^−/−^* mice. Unexpectedly, IL-1β treatments of WT mice resulted in increased permeability to biotin and elevated *C. rodentium* propagation within the lamina propria at 10 DPI, relative to untreated infected WT mice, in keeping with effects of IL-1β on histology and crypt hyperplasia in WT mice, as presented below. Together, these findings confirm the increased susceptibility of *Nlrp3^−/−^* mice to *C. rodentium* infection and also demonstrate the ability of IL-1β to at least partially reverse the effect of lacking NLRP3 activity on bacterial colonization and translocation, and barrier dysfunction. In addition, an appropriate IL-1β response was required for bacterial clearance as IL-1β treatments of infected WT mice increased bacterial infiltration, suggesting a detrimental role for excessive IL-1β.

### IL-1*β* treatments reduce *C. rodentium*-induced inflammation and modulate epithelial cell hyperplasia

**Figure 3:**
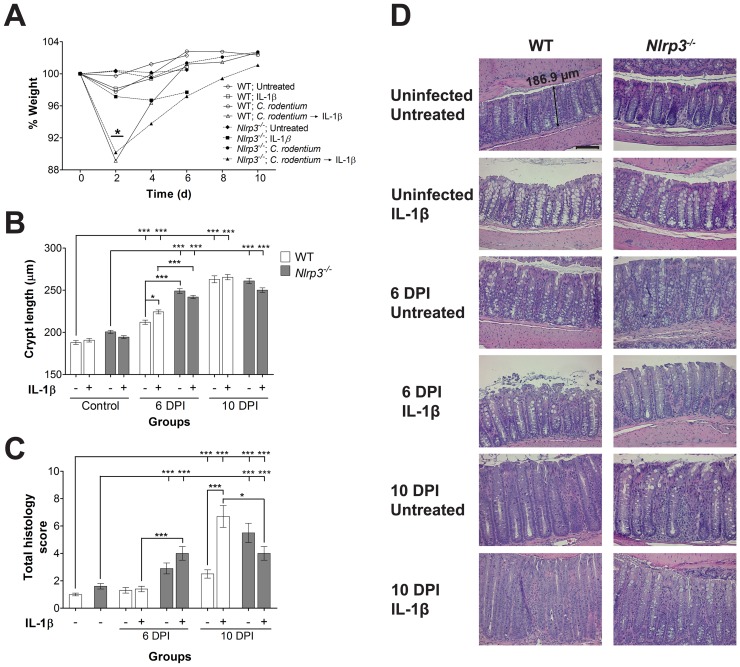
Colitis is modulated by IL-1β treatments. (A) IL-1β treatment of infected mice reduced WT and *Nlrp3^−/−^* body weights at 2 DPI compared to untreated mice, but weights later recovered (error bars not included for clarity); (B) Crypt lengths were measured using the ZEN software on Zeiss-acquired micrographs by an individual blinded to the identity of the slides. Crypt lengths increased significantly within 6 DPI in *Nlrp3^−/−^* mice compared to WT mice. IL-1β treatments increased the crypt lengths at 6 DPI in WT mice compared to untreated mice. Data represents mean per crypt ± SE; (C) Histological scores of colitis were assessed in a blinded fashion and graded as described previously [34]. Scoring revealed elevated severity of disease in IL-1β-treated WT mice at 10 DPI, while IL-1β-treated *Nlrp3^−/−^* mice had reduction in damage. Data represents mean ± SE; (D) H&E-stained colonic sections of untreated and treated mice. Increase in crypt lengths, loss of goblet cells, and cellular damage can be noted in infected mice (further details in **Figure S2**). Hematoxylin and eosin staining, magnification X200, bar 100 µm. One asterisk: *P*<0.05, three asterisks: *P*<0.001.

IL-1β treatments of infected WT and *Nlrp3^−/−^* mice significantly (*P*<0.05) reduced weights at 2 DPI compared to untreated mice (**Figure 3A**); however all groups retained body weights comparable to controls by 6 and 10 DPI. To determine the effects of IL-1β treatments on crypt hyperplasia, the lengths of colonic crypts (**Figure 3B**) as well as the cellular proliferation by staining for PCNA was measured (**Figure S1**). Crypt lengths increased significantly (*P*<0.001) within 6 DPI in *Nlrp3^−/−^* mice compared to infected WT mice; however crypt lengths were not different at 10 DPI. IL-1β treatments increased the crypt lengths at 6 DPI in WT mice significantly compared to untreated mice (*P*<0.001), while showing a trend for reduced crypt length in treated *Nlrp3^−/−^* mice. Cellular proliferation and quantification of PCNA-positive cells per crypt at 6 and 10 DPI showed significant increases in proliferation in WT and *Nlrp3^−/−^* mice (*P*<0.001; **Figure S1**). IL-1β treatments increased PCNA-positive cells at 6 DPI in WT mice significantly compared to untreated mice (*P*<0.001), suggesting that the increases in crypt lengths was due to cell proliferation and not reduced cell death.

IL-1β treatments of uninfected mice did not elicit any inflammatory damage. *C. rodentium* infection manifested with intestinal inflammation, colonic epithelial damage, and crypt abscesses (examples shown in **Figure S2**). These parameters were more predominant at 10 DPI as expressed in the total histology score (**Figure 3C**), and demonstrated by H&E staining (**Figure 3D and S** **2**). At 6 DPI, the histology scores in WT mice were not elevated in the IL-1β treated mice; however *Nlrp3^−/−^* mice exhibited an elevated histology score, and IL-1β treatments elevated this damage compared to WT mice (*P*<0.001). Interestingly at 10 DPI, the IL-1β treatments elevated the histology scores of WT mice (*P*<0.001); in comparison, IL-1β treatments mildly reduced the damage in *Nlrp3^−/−^* mice (*P*<0.05) compared to treated WT mice, in keeping with the protective effects this treatment had on colonization and integrity at later stages of infection.

### IL-1*β* treatments modulate cytokine secretion and augment macrophage tissue infiltration

**Figure 4:**
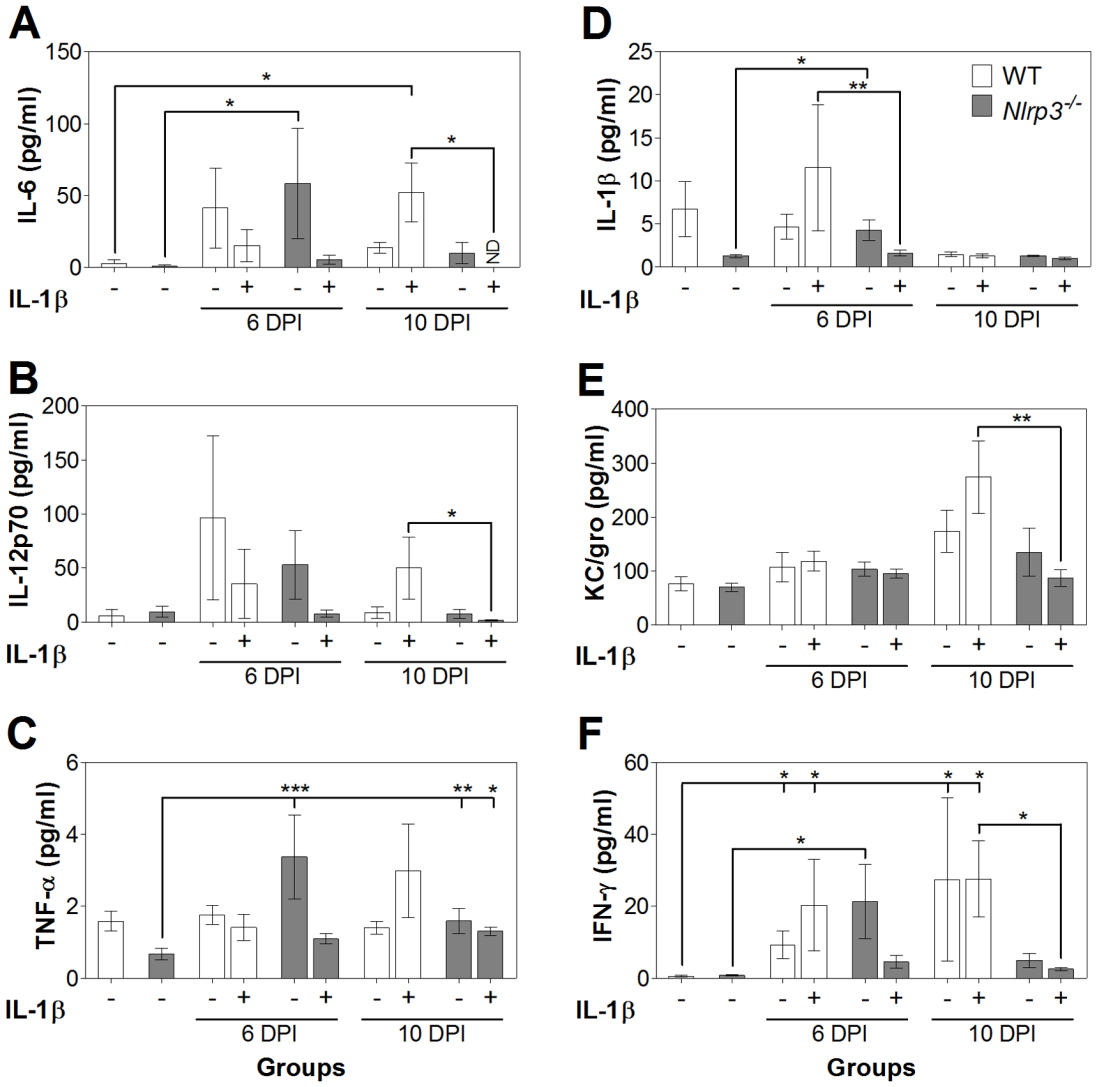
IL-1β treatments modulate the systemic cytokine response. IL-6 (A), IL-12p70 (B), TNF-α (C), IL-1β (D), KC/gro (E), and IFN-γ (F) were measured using ELISA-based assays. Generally, secretion of pro-inflammatory cytokines was dissipated in *Nlrp3^−/−^* mice at 10 DPI compared to WT mice. One asterisk: *P*<0.05, two asterisks: *P*<0.01, three asterisks: *P*<0.001; ND, not detected.

In order to better define the inflammatory milieu and how it is affected by NLRP3 and IL-1β, levels of pro-inflammatory cytokines in the plasma (**Figure 4**) and colon tissue (**Figure S3**) were determined by multiplex ELISA. Infection increased the levels of pro-inflammatory cytokines IL-6 and IFN-γ in WT mice and as well IL-1β in *Nlrp3^−/−^* mice. While the IL-1β treatments did not significantly affect cytokine levels compared to non-treated infection groups, IL-1β treatment of WT, compared to *Nlrp3^−/−^* mice elevated plasma cytokine levels of IL-1β at 6 DPI, and IL-6, IL-12p70, KC/gro, and IFN-γ at 10 DPI, suggestive of macrophage stimulation of the immune system. Of note, IL-1β injection did not increase measured levels of total IL-1β, indicating that this cytokine was likely eliminated by DPI 6. As well, IL-1β treatments increased colon tissue cytokine levels in *Nlrp3^−/−^* mice (**Figure S3**). Helper T-cell mediated IL-17 and IL-22 levels were not significantly elevated at DPI 6 or 10 (*P*>0.05; data not shown), and MCP-1 levels were not different than those measured in controls (*P*>0.05; data not shown).

**Figure 5:**
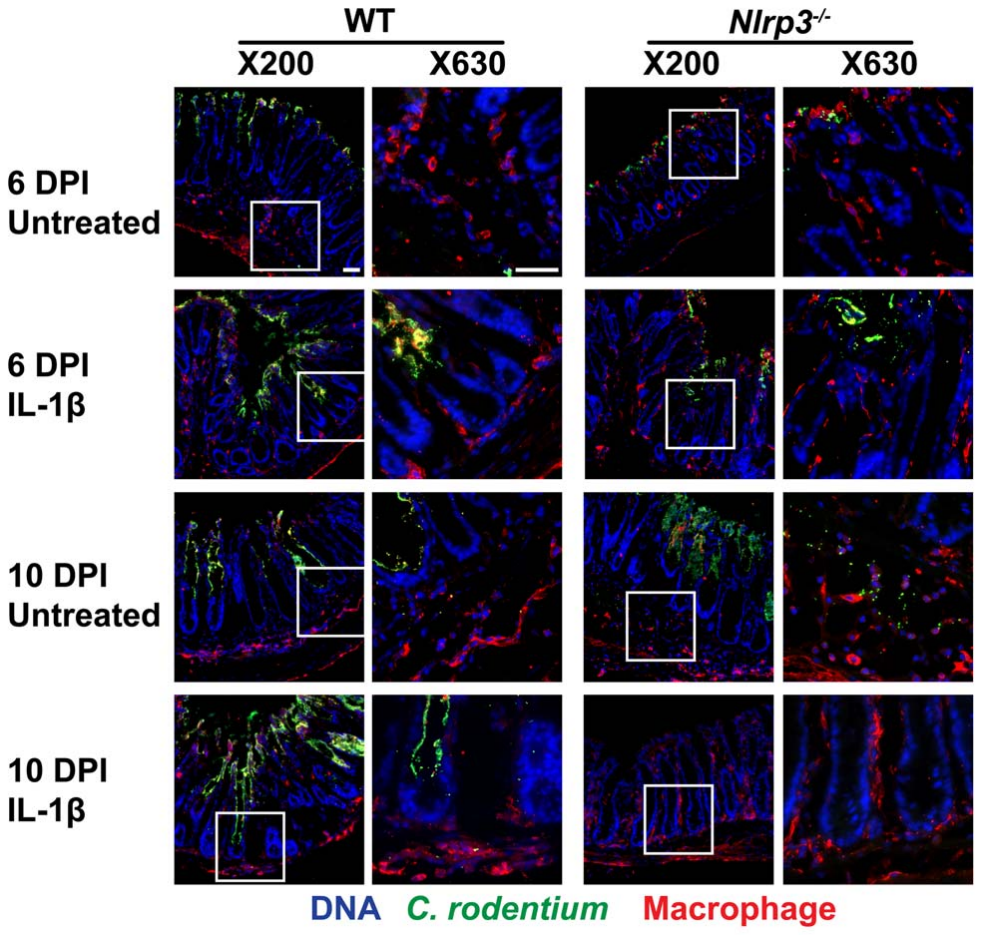
IL-1β treatments augment macrophage colonic infiltration. Macrophage infiltration was assessed by staining cryosections of distal colons. An increase in the number of macrophages (red; indicated by arrowhead in higher magnification panels on the left) in close proximity of *C. rodentium* (green) at 10 DPI in *Nlrp3^−/−^* mice is noted. With IL-1β treatments, the macrophages in crypts and mucosal lining appeared increased and bacterial infiltration into the crypts reduced. Bar 50 µm.

Macrophage infiltration at 6 DPI was restricted mostly to the submucosal regions of untreated WT mice and the mucosal surface in *Nlrp3^−/−^* mice (**Figure 5**). At 10 DPI, *C. rodentium* was detected within the mucosal lining with appropriate infiltration of macrophages in close proximity to invading bacteria in WT mice, while *Nlrp3^−/−^* mice failed to control bacterial dissemination. However, IL-1β treatments increased macrophage infiltration into the mucosa and lamina propria in WT mice, without restricting bacteria penetration, while in *Nlrp3^−/−^* mice a drastic reduction of *C. rodentium* and elevated infiltration by macrophages were noted. These findings suggest that IL-1β could drive macrophage infiltration and elimination of bacteria at 10 DPI, as seen in *Nlrp3^−/−^* mice; in contrast, while macrophages still infiltrate crypts on 10 DPI in the presence of IL-1β in WT mice, this does not result in effective clearance of bacteria.

### Exogenous IL-1*β* induce phagocytosis and breakdown of the epithelial barrier

**Figure 6:**
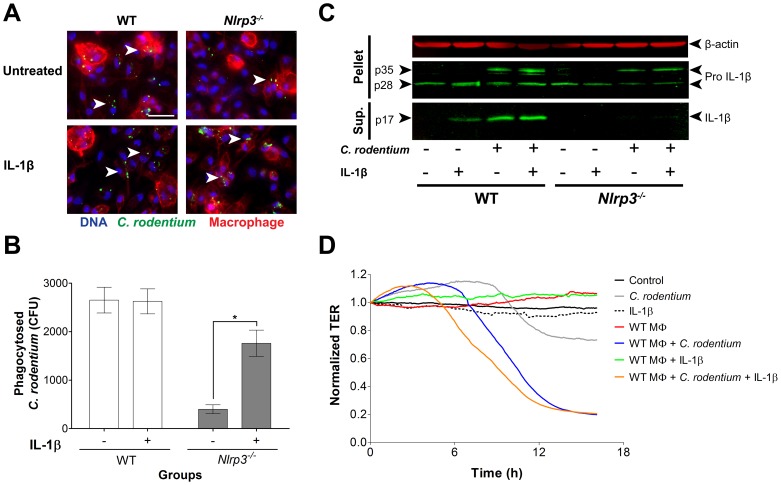
IL-1β enhances *C. rodentium* phagocytosis, while overcompensation of IL-1β augments epithelial barrier damage *in vitro*. (A) Exogenous IL-1β induced *C. rodentium* phagocytosis (arrowhead) by peritoneal macrophages *in vitro*, especially in *Nlrp3^−/−^* macrophages. Magnification X630, bar 50 µm; (B) Quantification showed an increase of intracellular *C. rodentium* in IL-1β treated *Nlrp3^−/−^* macrophages but no effect on WT macrophages. Data represents mean ± SE. One asterisk *P*<0.05; (C) Western blot analysis of mature IL-1β in the supernatant of peritoneal macrophages and pro-IL-1β in the cellular component; (D) The epithelial integrity of *C. rodentium*-infected CMT-93 cells assessed by ECIS, deteriorated in presence WT macrophages and even more with the addition of IL-1β. Data represents the mean of two independent experiments.

In keeping with our *in vivo* findings, indicating that lack of NLRP3 impedes macrophage function and bacterial uptake and clearance, and that complementation with IL-1β rescues this effect, we found that exogenous administration of IL-1β to peritoneal macrophages from *Nlrp3^−/−^* mice improved phagocytosis of *C. rodentium*, but not in macrophages from WT mice (**Figure 6A**). These findings were complemented with quantification of live intracellular *C. rodentium* on MacConkey agar plates after incubation of bacteria with macrophages and treatment with gentamicin to kill all extracellular bacteria, showing poor phagocytosis by macrophages from *Nlrp3^−/−^* mice, which are recovered with a 5-fold increase by IL-1β treatments, an effect absent in WT macrophages (**Figure 6B**). Western blot analysis (**Figure 6C**) reaffirmed that peritoneal macrophages from WT but not *Nlrp3^−/−^* mice secreted mature IL-1β to cell supernatants after infection with *C. rodentium*, and as well, that administration of exogenous IL-1β induced maturation of the cytokine in uninfected WT macrophages. *Nlrp3^−/−^* macrophages did, as expected, have pro-IL-1β (seen in cell pellets) but did not secrete mature IL-1β in presence of *C. rodentium* regardless of whether they were treated with exogenous IL-1β or not. This suggests that the majority of active IL-1β is produced through NLRP3 activity and not an accessory pathway in this model.

To define potential mechanisms for the observed detrimental overcompensation of IL-1β in treated WT mice, we examined the possibility that excessive infiltration by macrophages may have contributed to the damage to the epithelial barrier, in addition to infection. Using a polarized epithelial monolayer, we found that infection with *C. rodentium* on the apical surface had a minor effect on the TER, indicating partial barrier disruption. Macrophages or IL-1β treatment alone had no effect on the TER. Addition of WT macrophages to infected cells resulted in substantial and early descent of the TER and supplementing with IL-1β disrupted the barrier even further (**Figure 6D**). Macrophages lacking the NLRP3 inflammasome did not reduce the TER compared to *C. rodentium* infected cells; however the compensation of IL-1β to these macrophages deteriorated the TER (**Figure S4**). Overall, these results indicate that IL-1β compensation in mice lacking the NLRP3 inflammasome protects the host from *C. rodentium* infection by reducing colonization; however, overcompensation of this pathway in WT mice by excess IL-1β contributes to epithelial barrier damage.

## Discussion

This study was performed to better characterize the role of NLRP3 in early stages of infection and to determine if administration of IL-1β to *Nlrp3^−/−^* mice would bypass the lack of NLRP3 inflammasome. Such insights are required to define the roles of NLRP3 and IL-1β in host response to infection, and to establish a potential therapeutic application to this knowledge. Although the main focus of this study was the role of macrophages in the acute phase of *C. rodentium* infection, the importance of other immune regulatory cells and their relationship with macrophages should not be neglected [4]. Macrophage induction of CXCL-2, a ligand for the CXCR2 receptor, induces neutrophil influx and contributes to clearance of *C. rodentium* [15]. Colonic macrophages have also been shown to support innate lymphoid cells expressing the IL-22 cytokine to clear *C. rodentium* by increased expression of RegIII antimicrobial peptides [36], and exogenous RegIIIγ in *Il-22^−/−^* mice improves survival [37]. As well, the depletion of natural killer cells reduces infiltration of macrophages to the colon but elevates systemic *C. rodentium* dissemination [38]. An intricate system also exists between T helper cells and macrophages, as T helper IL-17 expression induces cytokine production in peritoneal macrophages and protects mice from *C. rodentium* infection [39].

The systemic administration of IL-1β has been shown to compensate its deficiency in skin lesions of *Il-1β^−/−^* mice, induced by *Staphylococcus aureus* infection [32]. Here we demonstrated that IL-1β production was required for effective macrophage responses to *C. rodentium*, and that excessive extraneous IL-1β in WT mice was deleterious, and exacerbated the infection. It is important to note that an activated caspase-1 is also required for IL-1β production and that, in keeping with our hypothesis, *Casp1^−/−^* mice (who also lack caspase-11) have an increased susceptibility to *C. rodentium* infection [30]. However *Casp1^−/−^* mice tend to have a higher severity of infection than *Nlrp3^−/−^* mice, possibly due to caspase-1′s broader involvement in processing of additional cytokines responsible for limiting intracellular pathogen replication [40], epithelial cell repair [41], and pyroptosis (an inflammatory form of cell death) [42]. In our study, we chose to use the *Nlrp3^−/−^* model since activation of the NLRP3 inflammasome specifically matures the IL-1β cytokine [19], and we assessed the effects of administered IL-1β in these mice, producing a more specific model.

*C. rodentium* colonization begins in the cecum (more specifically the cecal patch) and requires 3 to 5 days to spread to and colonize the colon [43]. We hypothesized that *Nlrp3^−/−^* mice may be more susceptible to *C. rodentium* infection due to reduced macrophage recruitment and response; therefore, IL-1β treatments in the early stages of the *C. rodentium* colonization in *Nlrp3^−/−^* mice were administered to stimulate macrophage response at the time of colonization. Treatments were then halted to determine whether macrophages may alleviate the infection or augment the inflammatory damage up to 10 DPI. Parallel to colonization, the eradication of *C. rodentium* also begins with the cecum before bacterial elimination from the colon [3]. This may explain the lowered *C. rodentium* colonization of the cecum in IL-1β-treated *Nlrp3^−/−^* mice at 6 DPI, and in the colon at 10 DPI seen in our study. Dissemination of *C. rodentium* to MLN was also lowered in IL-1β-treated *Nlrp3^−/−^* mice at 10 DPI. This is consistent with a recent study showing that administration of granulocyte-macrophage colony-stimulating factor (GM-CSF) to *GM-CSF^−/−^* mice protected them from *C. rodentium* dissemination at 2 weeks post-infection [44].

In order to define the functional implications of IL-1β activity in our model, we evaluated the effect of IL-1β on *C. rodentium* colonization by assessing epithelial barrier function. The importance of the NLRP3 inflammasome in epithelial integrity [29], and the disruption of the barrier and tight junctions leading to *C. rodentium* dissemination have been widely shown [13], [33], [45]. We showed that reduction in colonization and biotin permeability in the crypts of WT mice were exacerbated by IL-1β treatments. In *Nlrp3^−/−^* mice, we observed a reverse effect with IL-1β treatments alleviating *C. rodentium* colonization and biotin penetration, suggesting that a balance IL-1β response is required for effective barrier function as both excess and lack of IL-1β activity were detrimental. This observation is in keeping with observations in other host-microbe settings, such as *Mycobacterium* infections [46], and the ability of increased NLRP3-mediated production of IL-1β by macrophages to enhance inflammation and tissue damage [47], [48].

Crypt hyperplasia and epithelial damage are typical features of *C. rodentium* infection [6], and the deficiency of the NLRP3 inflammasome has been shown to augment these effects [19]. In keeping with our expectation, *C. rodentium* infection induced crypt hyperplasia in *Nlrp3^−/−^* mice higher than in WT mice, and IL-1β treatments had a reducing trend on crypt lengths in *Nlrp3^−/−^* mice. Models of experimental colitis have shown that macrophages promote crypt proliferation [49] and that NLRP3 inflammasome production of IL-18 (not IL-1β) is required for epithelial protection [29], [48], [50]. Lebeis *et al.* [31] have also reported that *Il-18^−/−^* mice survive *C. rodentium* infection in comparison to mice lacking the IL-1 receptor. Furthermore, recent evidence suggests that the NLRP3 inflammasome and its maturation of IL-18 downregulates the IL-22 binding protein, an inhibitor of IL-22, which promotes cell repair in inflammatory bowel diseases [51]. This could explain the inefficiency of IL-1β to modulate epithelial proliferation, and more so exacerbate the hyperplasia.

The histology severity score of infected *Nlrp3^−/−^* mice was higher compared to WT mice at 6 DPI; interestingly at 10 DPI, IL-1β treatment elevated the histology score in WT mice to levels even higher than in *Nlrp3^−/−^* mice. This trend was consistent with our findings for crypt hyperplasia, suggesting that enterocyte hyperplasia was an innate response to damage of the mucosal lining. The capacity of IL-1β to reduce disease severity in *Nlrp3^−/−^* mice was not surprising, as IL-1β is needed for reduction of intestinal damage [19], [31]. However, the exacerbation of disease in IL-1β-treated WT mice was interesting as it showed that the overcompensation of IL-1β was injurious. Indeed, hypersecretion of IL-1β in human macrophages due to polymorphic mutations in the *NLRP3* gene have been implicated in multiple autoinflammatory diseases, displaying the fine line between homeostasis and pathogenesis [52]–[55]. Particularly in Crohn disease, recent evidence suggests involvement of abnormal macrophage function with increased IL-1β secretion and impaired bacterial killing [56], [57]. Other investigators have shown that inflammatory macrophages contribute to severity of *C. rodentium* infection and damage to the mucosal lining [12].

The secretion of pro-inflammatory cytokines, including IL-1β, and macrophage infiltration play crucial roles in *C. rodentium* clearance [58]. An examination of a panel of pro-inflammatory cytokines in IL-1β treated groups, revealed increases in cytokines from infected WT mice compared to *Nlrp3^−/−^* mice, hinting at a higher inflammatory response and possibly greater inflammatory damage. IL-17 was not detected in our samples, possibly due to the early stage of infection [39], and IL-22 levels were not affected by NLRP3 or IL-1 β on DPI 6 or 10, as peak levels are typically noted at 4 days, and dissipate by 6 DPI, as observed by other investigators [59]. These findings do not exclude a role for these cytokines as they have been shown to be involved in earlier stages of infection [18], [37].

Finally, we sought to determine the effects of IL-1β on macrophage infiltration and phagocytosis. Deletion of the NLRP3 inflammasome did not restrict macrophage infiltration to the site of infection; however the *Nlrp3^−/−^* macrophages were unable to control *C. rodentium* colonization, leading to increases in bacterial loads in the lamina propria and translocation into the mucosa. IL-1β treatments reduced bacterial infiltration with increases of macrophages infiltrating into the lamina propria, suggesting that IL-1β might potentiate macrophage function. We confirmed this proposed effect by administration of exogenous IL-1β to peritoneal macrophages *in vitro*. WT macrophages were capable of phagocytosis, in the presence or absence of exogenous IL-1β, while phagocytosis was impaired in *Nlrp3^−/−^* macrophages, and the administration of IL-1β effectively restored this function. Western blot analysis of WT macrophages showed that the mature IL-1β cytokine was secreted in the presence of administered IL-1β or *C. rodentium*, both absent in *Nlrp3^−/−^* macrophages. To elucidate why IL-1β-treated WT mice were more susceptible to epithelial injury, a comparative *in vitro* model of epithelial barrier function was used. When polarized epithelial cells where inoculated with *C. rodentium*, there was minimal epithelial damage; however, the addition of WT macrophages and furthermore the overcompensation of IL-1β damaged the barrier, representing the detrimental potential of macrophages, especially in the presence of excessive IL-1β. This provides an important insight into the role of macrophages and the balance of IL-1β in managing the infection.

Taken together, these findings indicate that a delicate IL-1β cytokine balance is required to promote effective clearance of an acute *C. rodentium* infection in *Nlrp3^−/−^* mice. Moreover, overcompensation of IL-1β in this system seems to be detrimental to WT mice and induces colonic damage. This study further supports the importance of the NLRP3 inflammasome and macrophages in enteric infections as well as inflammatory diseases and offers potential innovative approaches for manipulating these pathways to control gut inflammation.

## Supporting Information

Figure S1 **IL-1β treatments augment colonic epithelial cell hyperplasia.** (A) PCNA- (red; representing proliferating cells) and cellular DNA- (blue; DAPI) stained colonic sections of untreated and treated mice, showing increased proliferation in all infected mice. (B) PCNA-positive cells were counted using the ZEN 2012 software by an individual blinded to the study, documenting the increase in proliferation of *Nlrp3^−/−^* and WT mice, without obvious effect for IL-1β. Data represents mean per crypt ± SE; magnification X200, bar 100 µm. One asterisk *P*<0.05.
(TIF)

Figure S2 **IL-1β treatments reduce colonic damage in** ***Nlrp3^−/−^*** **mice.** Representative micrographs of distal colons of WT and *Nlrp3^−/−^* mice: (A) 10 DPI WT mice: the colonic architecture is intact with a progressive maturation of enterocytes; (B) 10 DPI IL-1β-treated WT mice: the crypt architecture is mildly distorted (asterisk) with intracryptal neutrophils (arrowheads) and moderate inflammation in the lamina propria (arrows); (C) 10 DPI *Nlrp3^−/−^* mice: crypt architecture distortion is noted (asterisk), with marked inflammation, paucity of goblet cells (arrowhead), and neutrophils in the interstitium (arrow) and infiltrating the crypts; (D) 10 DPI IL-1β-treated *Nlrp3^−/−^* mice: the morphology of the intestinal architecture is partially restored with normal maturation of the intestinal cells, although there is focally some increase of cellularity (arrow) between the ordered crypts. Magnification X200, Hematoxylin and eosin staining.
(TIF)

Figure S3 **IL-1β treatments modulate colonic tissue cytokine response.** Proinflammatory cytokines IL-6 (A), IL-12p70 (B), TNF-α (C), IL-1β (D), KC/gro (E), and IFN-γ (F) were measured in colon homogenates by a multiplex ELISA-based assay. Generally, the secretion of some pro-inflammatory cytokines in the colon tissue was elevated at 6 DPI in *Nlrp3^−/−^* mice compared to WT mice. One asterisk *P*<0.05.
(TIF)

Figure S4 **IL-1β compensation in** ***Nlrp3^−/−^*** **macrophages augments epithelial barrier damage** ***in vitro*** **.** The epithelial barrier of *C. rodentium*-infected CMT-93 cells assessed by ECIS were not considerably damaged in presence of macrophages, however the compensation of IL-1β damaged the barrier. This reflects the detrimental effect of IL-1β on macrophages, which is seen *in vitro* even in cells from *Nlrp3^−/−^* mice, possibly due to lack of regulatory pathways in this simplified model. Data represents the mean of two independent experiments.
(TIF)

Appendix S1 **Supplementary data.**
(DOCX)
