# Supplementary figures and images for: A Balanced IL-1β Activity Is Required for Host Response to Citrobacter rodentium Infection
Source: PLoS One. 2013 Dec 2;8(12):e80656. doi: 10.1371/journal.pone.0080656 (PMC3846666; doi:10.1371/journal.pone.0080656)

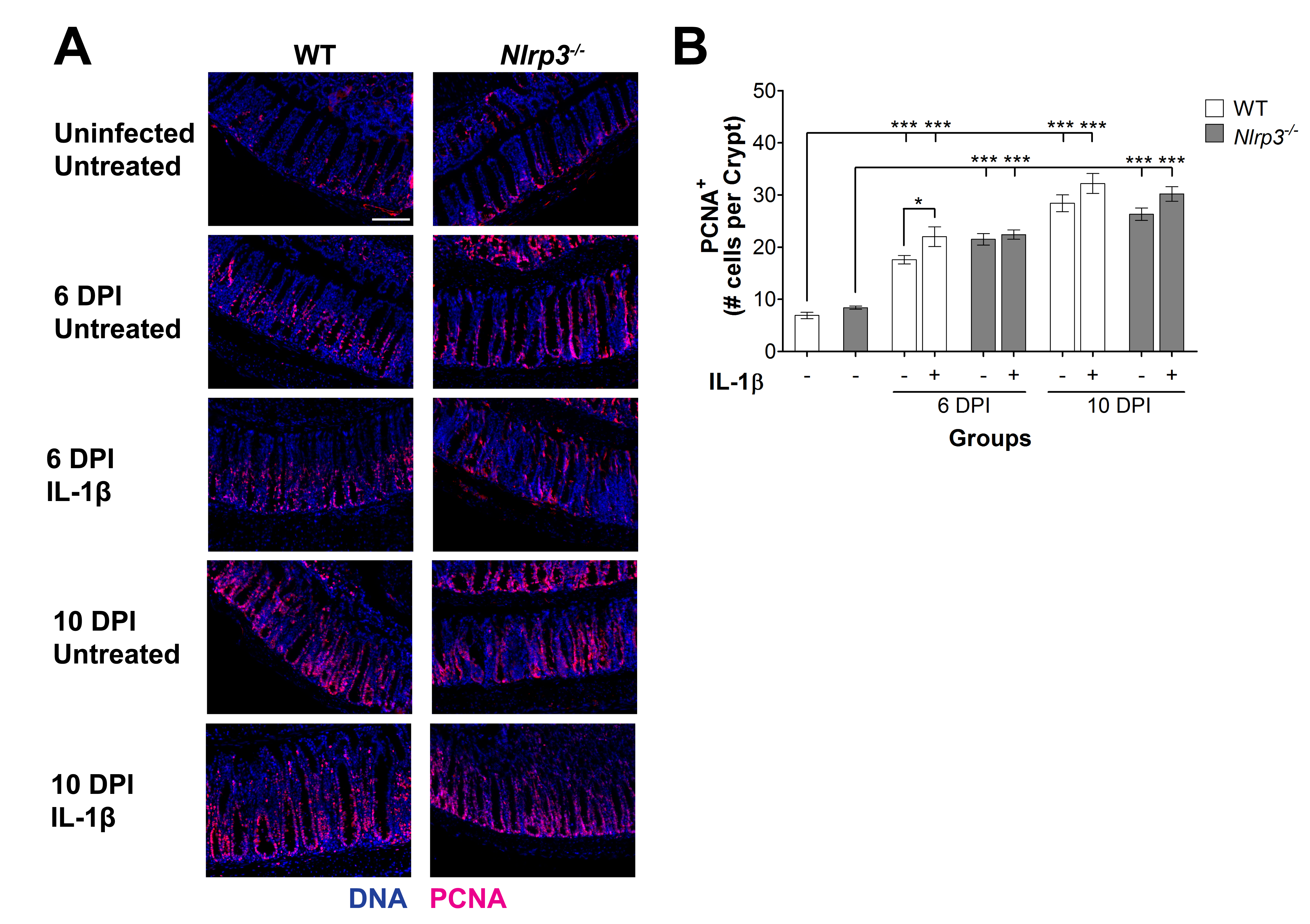

Supplement: Figure S1 — IL-1β treatments augment colonic epithelial cell hyperplasia. (A) PCNA- (red; representing proliferating cells) and cellular DNA- (blue; DAPI) stained colonic sections of untreated and treated mice, showing increased proliferation in all infected mice. (B) PCNA-positive cells were counted using the ZEN 2012 software by an individual blinded to the study, documenting the increase in proliferation of Nlrp3−/− and WT mice, without obvious effect for IL-1β. Data represents mean per crypt ± SE; magnification X200, bar 100 µm. One asterisk P<0.05. (TIF) [file pone.0080656.s001.tif]

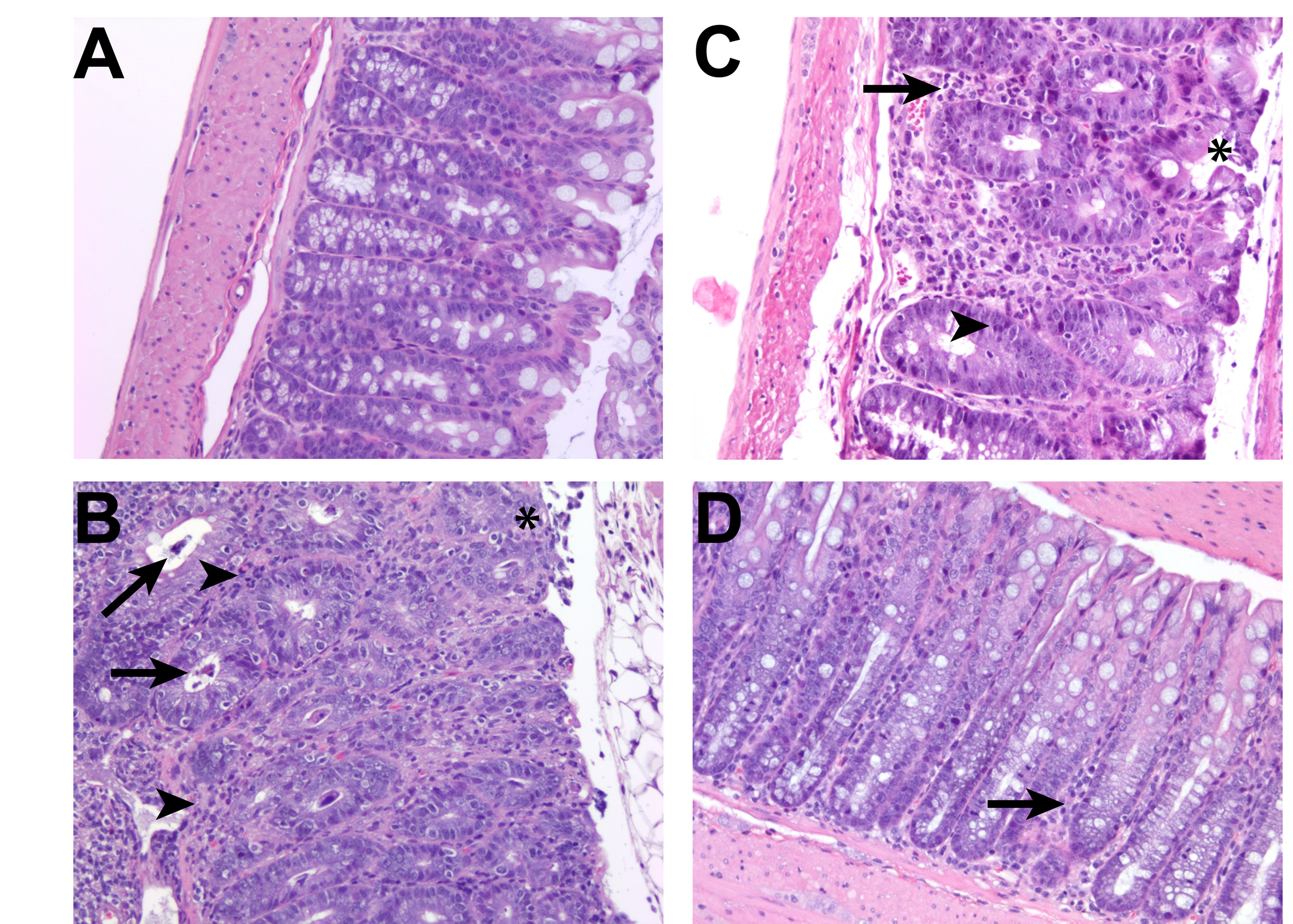

Supplement: Figure S2 — IL-1β treatments reduce colonic damage in Nlrp3−/− mice. Representative micrographs of distal colons of WT and Nlrp3−/− mice: (A) 10 DPI WT mice: the colonic architecture is intact with a progressive maturation of enterocytes; (B) 10 DPI IL-1β-treated WT mice: the crypt architecture is mildly distorted (asterisk) with intracryptal neutrophils (arrowheads) and moderate inflammation in the lamina propria (arrows); (C) 10 DPI Nlrp3−/− mice: crypt architecture distortion is noted (asterisk), with marked inflammation, paucity of goblet cells (arrowhead), and neutrophils in the interstitium (arrow) and infiltrating the crypts; (D) 10 DPI IL-1β-treated Nlrp3−/− mice: the morphology of the intestinal architecture is partially restored with normal maturation of the intestinal cells, although there is focally some increase of cellularity (arrow) between the ordered crypts. Magnification X200, Hematoxylin and eosin staining. (TIF) [file pone.0080656.s002.tif]

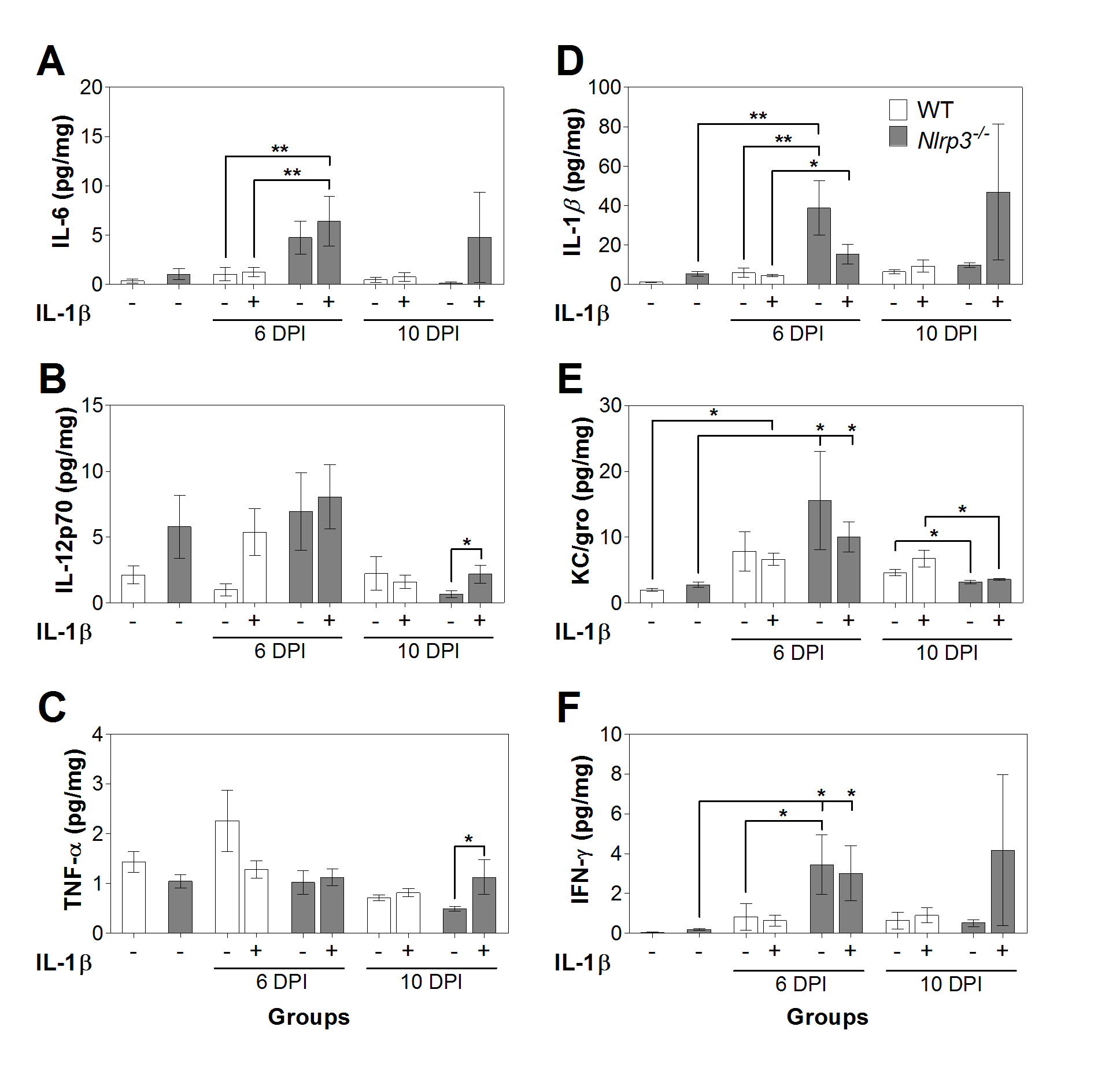

Supplement: Figure S3 — IL-1β treatments modulate colonic tissue cytokine response. Proinflammatory cytokines IL-6 (A), IL-12p70 (B), TNF-α (C), IL-1β (D), KC/gro (E), and IFN-γ (F) were measured in colon homogenates by a multiplex ELISA-based assay. Generally, the secretion of some pro-inflammatory cytokines in the colon tissue was elevated at 6 DPI in Nlrp3−/− mice compared to WT mice. One asterisk P<0.05. (TIF) [file pone.0080656.s003.tif]

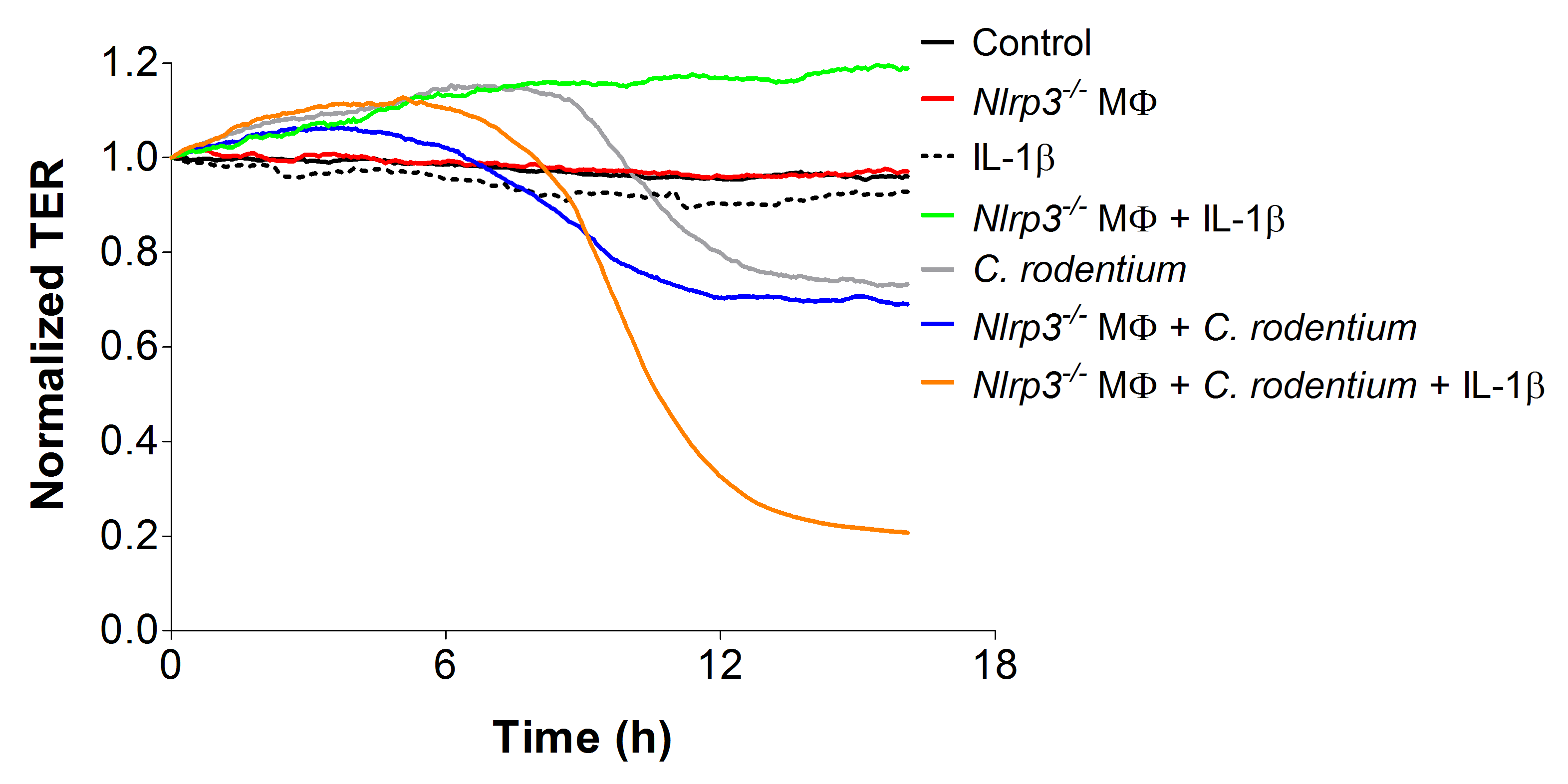

Supplement: Figure S4 — IL-1β compensation in Nlrp3−/− macrophages augments epithelial barrier damage in vitro . The epithelial barrier of C. rodentium-infected CMT-93 cells assessed by ECIS were not considerably damaged in presence of macrophages, however the compensation of IL-1β damaged the barrier. This reflects the detrimental effect of IL-1β on macrophages, which is seen in vitro even in cells from Nlrp3−/− mice, possibly due to lack of regulatory pathways in this simplified model. Data represents the mean of two independent experiments. (TIF) [file pone.0080656.s004.tif]
