# Supplementary material for: A Balanced IL-1β Activity Is Required for Host Response to Citrobacter rodentium Infection
Source: PLoS One. 2013 Dec 2;8(12):e80656. doi: 10.1371/journal.pone.0080656 (PMC3846666; doi:10.1371/journal.pone.0080656)
Supplement: Appendix S1 — Supplementary data. (DOCX) [file pone.0080656.s005.docx]

**SUPPLEMENTARY DATA**

**RT-PCR**

RT-PCR was performed following the manufacturer’s protocol (QuantiFast SYBR Green RT-PCR kit; Qiagen, Toronto, ON, Canada). *C. rodentium* *espB* (a type III secretion system effector protein) primers used were Forward: 5’-GCTTCTGCGAAGTCTGTCAA-3’ and Reverse: 5’-CAGTAAAGCGACTTAACAGATT-3’ (Integrated DNA Technologies, Toronto, ON, Canada). RT-PCR of bacterial colonies in non-infected mice was *C. rodentium* negative.

**Cellular proliferation**

To determine the colonic epithelial proliferation, paraffin-embedded sections were used based on a previously published method [[1](#_ENREF_1)]. In brief, slides were deparaffinised by microwaving for 90 s and immersed in two changes of xylenes for 5 min, followed by rehydration in 100%, 90%, and 70% ethanol, and then water for 5 min each. Slides were immersed in a citrate buffer (10 mM, pH 6.0) with 0.05% Tween-20, and heated at 95°C for 15 min for antigen retrieval. Slides were washed with PBS-Tween 0.2% and blocked for 1 h at RT, and then stained with rabbit anti-proliferating cell nuclear antigen (PCNA, 1:250 dilution, overnight, 4°C; Abcam, Cambridge, MA, USA) followed by secondary anti-rabbit Alexa-546 (1:600 dilution, 1 h, RT; Life Technologies). Then, sections were counterstained with DAPI (5 µg/ml, 1 min, RT) and imaged at X200 magnification (Zeiss microscope). To quantify epithelial hyperplasia, PCNA positive cells per well-oriented crypts were counted by an individual blinded to the origin of each slide.

**Macrophage Isolation**

Peritoneal macrophages were induced by 1 ml intraperitoneal injection of 3% Thioglycollate Medium Brewer Modified (Fisher Scientific; Ottawa, ON, Canada) and macrophages were harvested 4 d later by peritoneal lavage. Cells were plated in 24-well plates in DMEM/F12, supplemented with 10% FBS, 2 mM L-glutamine, 100 U/ml penicillin, and 100 µg/ml streptomycin for 1 h. Non-adherent cells were washed off and macrophages were incubated overnight. For the ECIS experiments, peritoneal macrophages were collected in DMEM/F12, supplemented with 5% FBS, 2 mM L-glutamine, and 100 µg/ml gentamicin, washed thrice for 10 min at 500 g, and used immediately.

**Western Blot**

WT and *Nlrp3^-/-^* peritoneal macrophages were treated as described in the Materials and Methods section. Proteins in supernatants were precipitated by 20% TCA, centrifuged, washed with acetone, and resuspended in SDS loading buffer. The macrophages were lysed in M-PER reagent with protease inhibitor, and protein in supernatants precipitated as above. Recombinant IL-1β was also used to confirm the active form (data not shown). The protein was fractionated on 12% SDS-PAGE and transferred to a nitrocellulose membrane. The membrane was blocked in Odyssey blocking solution (1 h, RT), and incubated with primary rabbit anti-IL-1β (Abcam), and secondary donkey anti-rabbit IRDye 800CW (Mandel Scientific Company, Inc., Guelph, ON, Canada). For normalization against a housekeeping protein, the membrane was incubated with primary mouse anti-β-actin (Life Technologies), and secondary goat anti-mouse IRDye 680CW. The membrane was scanned using Li-Cor Odyssey (Mandel Scientific Company, Inc.).

**REFERENCE**

1. Dupaul-Chicoine J, Yeretssian G, Doiron K, Bergstrom KS, McIntire CR, et al. (2010) Control of intestinal homeostasis, colitis, and colitis-associated colorectal cancer by the inflammatory caspases. Immunity 32: 367-378.
